# Incidence and independent risk factors for heart failure in a population-based cohort of 19,892 individuals with newly diagnosed diabetes

**DOI:** 10.1007/s00125-026-06823-1

**Published:** 2026-08-03

**Authors:** Carl Lejonberg, Tomasz Czuba, Louise Bennet, Daniel E. Coral, Oscar O. Braun, Olle Melander, Maria F. Gomez, Emma Ahlqvist, J. Gustav Smith

**Affiliations:** 1 Department of Cardiology, Clinical Sciences, Lund University, Lund, Sweden; 2 Wallenberg Center for Molecular Medicine, Lund University, Lund, Sweden; 3 Lund University Diabetes Center, Lund University, Lund, Sweden; 4 The Wallenberg Laboratory/Department of Molecular and Clinical Medicine, Institute of Medicine and Science for Life Laboratory, Gothenburg University, Gothenburg, Sweden; 5 Department of Clinical Sciences, Lund University, Malmö, Sweden; 6 Diabetic Complications Unit, Department of Clinical Sciences in Malmö, Lund University Diabetes Center, Malmö, Sweden; 7 Department of Heart Failure and Valvular Disease, Skåne University Hospital, Lund, Sweden; 8 Department of Internal Medicine, Lund University, Malmö, Sweden; 9 Department of Cardiology, Sahlgrenska University Hospital, Gothenburg, Sweden

**Keywords:** Incident heart failure, Insulin resistance, SCORE2-Diabetes, Type 2 diabetes

## Abstract

**Aims/hypothesis:**

Heart failure (HF) is a common complication of diabetes, associated with a high mortality rate and a substantial healthcare burden. Residual HF risk persists despite achieving current treatment targets, suggesting a role for additional mechanisms beyond conventional risk factors. We sought to prospectively assess HF incidence and independent risk factors in individuals with new-onset diabetes.

**Methods:**

We used a prospective inception cohort (ANDIS, *n*=19,892) that included all individuals with newly diagnosed diabetes from southern Sweden between 2008 and 2021. Baseline clinical, biochemical and questionnaire data were linked to the Region Scania Care Database to obtain data on cardiovascular events and to the National Diabetes Registry to gather further data on cardiovascular risk factors that were not collected in the ANDIS study protocol. All-cause HF included all cases of HF regardless of underlying aetiology, whereas non-ischaemic HF was defined as HF occurring in individuals without prior acute myocardial infarction at baseline or during follow-up before the HF diagnosis. Cardiovascular disease incidence was estimated using the Kaplan–Meier method, and the associations with risk factors and incident HF were assessed using Cox proportional hazards regression.

**Results:**

The median age of the overall population was 62.5 years (IQR 52.6–70.3), while the median age among individuals who developed incident all-cause HF was 69.8 years (IQR 62.7–76.7). A total of 819 individuals (4.1%) with prevalent HF were excluded. During up to 14 years of follow-up (median 5.0), the cumulative incidence of HF was 4.8%, exceeding that of myocardial infarction (3.0%). No individuals with type 1 diabetes developed HF, but the incidence was similar across those with type 2 diabetes, latent autoimmune diabetes in adults, and secondary diabetes. A lower eGFR and higher HOMA2-IR, were associated with increased risk of all-cause and non-ischaemic HF, independently of the presence of conventional risk factors. Insulin resistance predicted all-cause HF independently of the established SCORE2-Diabetes risk score. The hazard ratio (HR) per 1 SD increase in HOMA2-IR was highest in the low-risk group (HR 1.89; 95% CI 1.02, 3.48; *p*=4.3 × 10^−2^), with concordant results observed for non-ischaemic HF.

**Conclusions/interpretation:**

The HF incidence was high, with approximately 1 in 20 individuals developing HF within 5 years after diagnosis of diabetes other than type 1, highlighting a notably high risk of developing HF soon after diabetes diagnosis. Beyond conventional risk factors, higher HOMA2-IR and lower renal function, but not hyperglycaemia or autoantibodies, were independent risk factors for HF. Measuring HOMA2-IR also provided prognostic information in addition to that provided by the SCORE2-Diabetes risk score, supporting its value in HF risk stratification.

**Graphical Abstract:**

**Figure:**
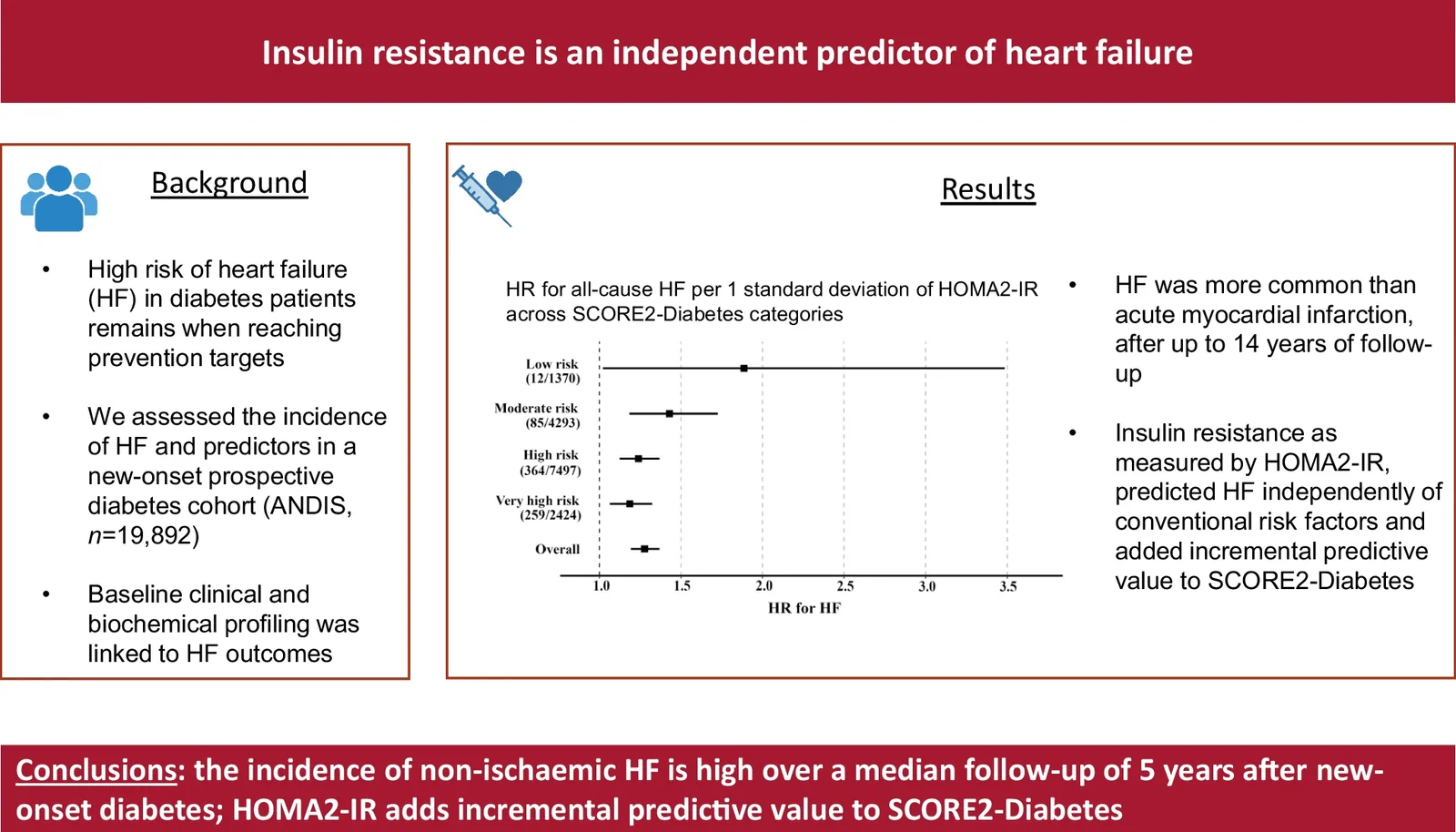

**Supplementary Information:**

The online version of this article (https://doi.org/10.1007/s00125-026-06823-1) contains peer-reviewed but unedited supplementary material.

**Figure:**
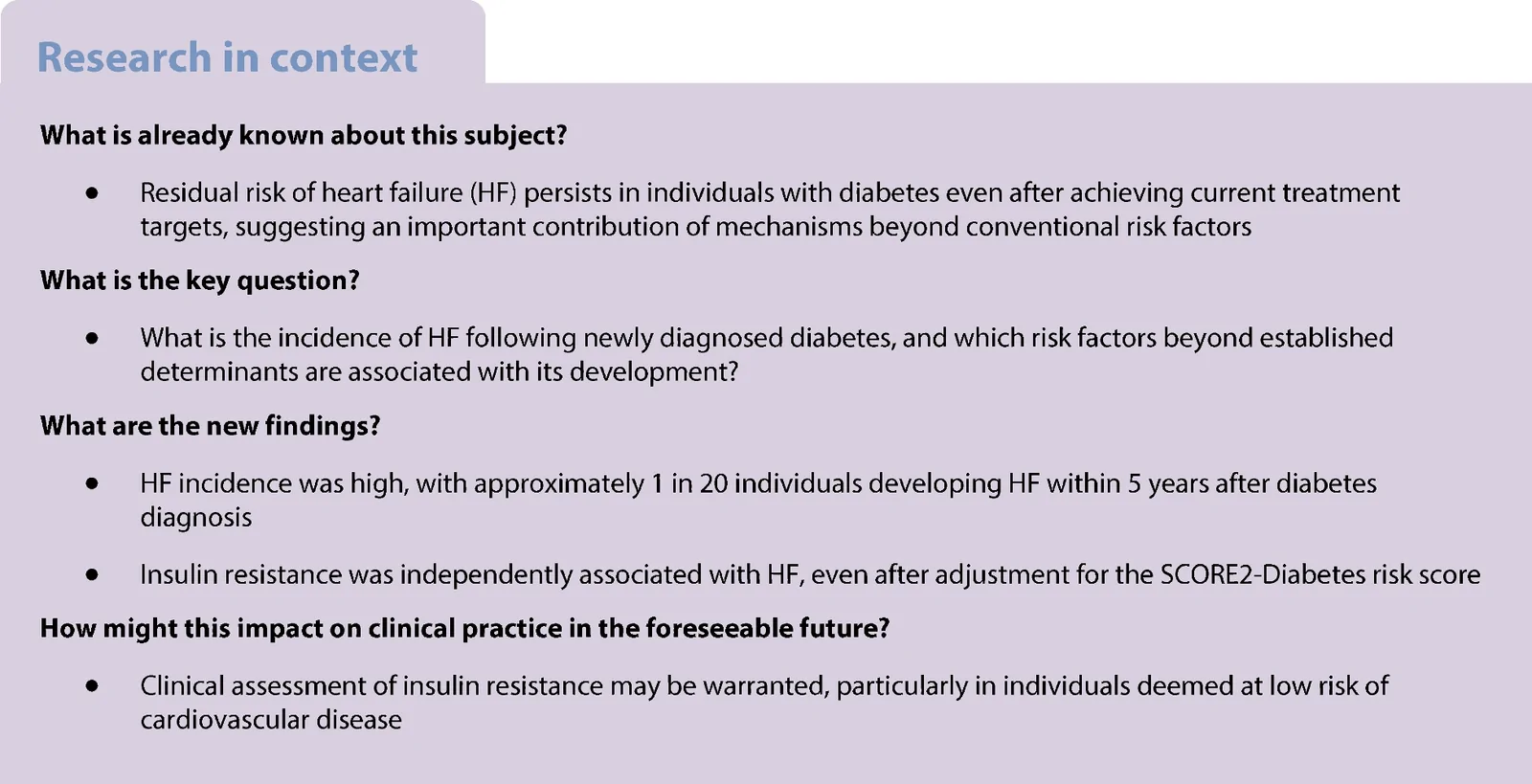

## Introduction

Type 2 diabetes, which accounts for 90–95% of all cases of diabetes mellitus [1], confers a two- to fourfold greater risk of cardiovascular events and death compared with the general population [2]. Successful treatment of conventional cardiovascular risk factors in individuals with type 2 diabetes, when achieved, results in little or no excess risk for death, myocardial infarction or stroke [2]. However, excess risk for heart failure (HF) remains markedly elevated [2], and confers substantial comorbidity and impairment of quality of life [3]. This observation suggests that pathophysiological mechanisms other than conventional risk factors contribute to incident HF in individuals with type 2 diabetes.

Heart failure is a well-established complication of diabetes [4–8]. In the mid-1950s, the increased cardiovascular risk in individuals with diabetes was conceived of as ‘diabetic angiopathy’ [9]. In 1972, the term ‘diabetic cardiomyopathy’ was devised to explain ventricular hypertrophy in individuals with diabetes and renal failure in the absence of major coronary artery disease [10]. The importance of symptomatic HF in type 2 diabetes was further emphasised in 1974, when the Framingham Heart Study [11] demonstrated an up to fivefold higher risk of HF in individuals with diabetes after adjustment for age, hypertension and coronary heart disease. To date, however, limited information is available on the incidence and determinants of HF in individuals with diabetes from the general population from the time of initial diagnosis [12–14]. Further, no uniform definition of diabetic cardiomyopathy [15] exists, it is not included in contemporary cardiomyopathy guidelines [16], and it remains unclear whether it represents a distinct clinical entity or not.

Importantly, the effects of glucose-lowering medications on the HF outcome have also been understudied in cardiovascular outcomes trials in individuals with diabetes. Heart failure has typically been omitted from the composite cardiovascular endpoint used in diabetes trials (known as ‘three-point major adverse cardiovascular events’ [3P-MACE]), which comprises cardiovascular death, non-fatal acute myocardial infarction (AMI) and non-fatal stroke. Concerns have been raised that 3P-MACE does not capture this important diabetes-related complication [4, 17, 18].

We therefore sought to determine the incidence and risk factors for pre-existing and new-onset HF (ischaemic as well as non-ischaemic) after initial diabetes diagnosis using a prospective inception cohort. Furthermore, we aimed to assess the ability of the SCORE2-Diabetes cardiovascular disease prediction algorithm to predict HF in individuals with diabetes. While this has previously been demonstrated for all-cause HF [19], we aimed to investigate whether the algorithm could predict both all-cause and non-ischaemic HF, and to assess whether use of measures of insulin resistance could add predictive value to the algorithm.

## Methods

### Study design and participants

The All New Diabetics in Scania (ANDIS) project is a prospective study within Scania county in southern Sweden (current population 1.4 million inhabitants) that aims to include all new clinically diagnosed cases of diabetes, as described previously [20]. Since the start of the project in 2008, diabetes nurses at each healthcare centre have obtained peripheral venous blood samples, anthropometric data, blood pressure measurements and questionnaire data within 1 year after initial diabetes diagnosis. Blood samples have been submitted to a central laboratory for analysis of several biomarkers, including fasting blood sugar, HbA_1c_, C-peptide and glutamic acid decarboxylase antibodies (GADA) using clinically approved assays. The eGFR was calculated using the CKD-EPI formula [21]. For each participant, insulin resistance was also measured using HOMA2-IR, based on fasting plasma glucose and C-peptide concentrations and calculated using the HOMA2 calculator as described previously [20].

Between 1 January 2008 and 31 December 2021, a total of 25,252 individuals 18 years or older with diabetes were included (>90% participation rate). The time from diabetes diagnosis to inclusion in ANDIS was defined as either time from diabetes diagnosis to the date of blood sampling or registration in the ANDIS database, whichever came first. Individuals who were included more than 1 year after diabetes diagnosis were excluded (*n*=93); the median time from diabetes diagnosis to inclusion in ANDIS was 0.1 years (IQR 0.04–0.25). A total of 19,892 individuals remained for analysis after exclusion of individuals without blood samples (*n*=5197), those with blood samples drawn more than 1 year before registration in ANDIS (*n*=9), those with a missing date for diabetes diagnosis (*n*=15), and those diagnosed with HF after diabetes diagnosis but prior to the sample date (*n*=46).

Sex was self-reported. Although information on country of birth is collected within the ANDIS cohort, detailed data on ethnicity and socioeconomic characteristics were not available for the present study.

The study was approved by the regional ethics review committee in Lund (584/2006, 2012/676 and 2017/904) and by the Swedish Ethical Review Authority (2023/08012).

### Definition of diabetes subtypes and HF

Type 1 diabetes was defined by GADA positivity together with a C-peptide concentration <0.3 nmol/l, while latent autoimmune diabetes in adults (LADA) was defined by GADA positivity and C-peptide concentrations ≥0.3 nmol/l. Secondary diabetes referred to diabetes that was attributable to another identifiable medical condition as assessed by the responsible physician. Other cases were defined as type 2 diabetes.

All-cause HF was defined using data from a region-wide register as described below, and thus was not divided into any aetiological or functional subgroups, whereas non-ischaemic HF was defined as HF occurring in the absence of a prior AMI.

### Cardiovascular disease outcomes

Information on cardiovascular events before the ANDIS inclusion date (prevalent cases) or after the ANDIS inclusion date (incident cases) was obtained from the Region Scania Care Database, which includes all diagnoses from Swedish hospitals, classified according to the International Classification of Disease (ICD) system, for inpatient care since 1987 and outpatient care since 2001. ICD-9 was used in Sweden between 1987 and 1997 and ICD-10 has been used since 1997. HF was defined based on code 428 for ICD-9, and codes I50, I11.0, I13.0, I13.2, I25.5, I42 and I43 for ICD-10. AMI was defined based on codes 410 and 412 for ICD-9, and codes I21 and I22 for ICD-10. Ischaemic stroke was defined based on codes 434 and 436 in ICD-9, and code I63 for ICD-10. Atrial fibrillation was defined based on code 427D in ICD-9 and code I48 in ICD-10.

The validity of diagnoses based on ICD codes in the Region Scania Care Database has previously been studied, showing high positive predictive values (85–95%) for most diagnoses [22]. An analysis of a primary diagnosis of HF in the Swedish hospital discharge register showed a validity of 95% based on criteria established by the European Society of Cardiology [23].

Information on all-cause and cardiovascular mortality for ANDIS participants was obtained from the Swedish Cause of Death Register, which records the date and cause of death for all Swedish citizens, until 3 December 2021. Cardiovascular mortality was defined as an underlying cause of death from any I code according to ICD-10, and secondarily in separate analyses that also included cardiovascular disease as a contributing cause of death.

### Cardiovascular risk factors

Additional information on several cardiovascular risk factors was obtained from the Swedish National Diabetes Register (NDR), including blood pressure, treatment for hypertension, cholesterol levels, lipid-lowering therapy and smoking. The NDR is a quality-of-care registry that was launched in 1996 with the intention of promoting evidence-based development of diabetes care [24]. Following an implementation project in primary care between 2002 and 2005, coverage of the register increased to 75% of individuals with diabetes nationally [24], and current coverage has been estimated by the Swedish Board of Health and Welfare to be 85% for adults and 98% for children (https://ndr.registercentrum.se/in-english/the-swedish-national-diabetes-register/p/BJyILnupj, accessed date 22 May 2024). The median time from diabetes diagnosis to availability of the NDR data used for analysis was 41 days (IQR 16–89 days).

### Derivation of the SCORE2-Diabetes risk score and prediction of HF

The SCORE2-Diabetes algorithm is calibrated to estimate the 10-year risk of non-fatal myocardial infarction, non-fatal stroke and cardiovascular death [25]. Recently, it was also demonstrated that the SCORE2-Diabetes risk score predicts HF and atrial fibrillation in individuals with newly diagnosed type 2 diabetes, based on data from the NDR [19]. In that study and the present study, individuals younger than 40 years and those with established cardiovascular disease at baseline (defined as prior myocardial infarction or stroke) were excluded, in accordance with European Society of Cardiology guidelines [25]. As the NDR is a nationwide register, our regional cohort constitutes a subset of this population, and partial overlap between the study populations is therefore expected. Individual 10-year risk was categorised as low (<5%), moderate (5% to <10%), high (10% to <20%) or very high (≥20%).

### Statistical analysis

Baseline characteristics are presented as means ± SD or median (IQR). In the ANDIS cohort, missing data were minimal (0.1–4.9% among included covariates). Missingness in the NDR registry was more common, and for this reason, variables with more than 50% missing data (urinary albumin–creatinine ratio and urinary albumin) were excluded. For the remaining variables, we tested for missingness completely at random (MCAR) using Little’s MCAR test, which is a χ^2^-based likelihood ratio statistic [26]. For variables fulfilling MCAR criteria, we performed random forest imputation [27]. Insulin resistance (as assessed using HOMA2-IR) displayed a skewed distribution, and was therefore natural log-transformed and standardised. There were two extreme HOMA2-IR outliers, which were interpreted as biologically implausible and incorrectly entered. These were excluded from non-imputed data analyses and treated as missing for imputation. BMI was dichotomised into obese (BMI ≥30 kg/m^2^) or non-obese (BMI <30 kg/m^2^).

Cardiovascular disease incidence from baseline was estimated by the Kaplan–Meier method, and crude incidence rates per 1000 person-years were calculated for each cardiovascular outcome stratified by age tertiles. The association of risk factors at baseline with incident all-cause HF and non-ischaemic HF (no prior history of AMI) was tested in individuals without prevalent HF using Cox proportional hazards regression, with censoring at death or end of follow-up. Separate analyses were performed using minimally adjusted models (age and sex) and multivariable-adjusted models. Cox proportional hazards models with non-imputed data were used for the primary analysis, and with imputed data included in the secondary analysis.

Potential non-linear associations between incident HF and glucose-related continuous variables (blood sugar, HbA_1c_ and C-peptide) were explored with restricted cubic splines using knot placements as proposed by Harrell et al, and centred around the mean values [28]. To assess whether insulin resistance could provide prognostic information beyond that provided by the SCORE2-Diabetes calculation, we estimated the hazard ratio (HR) per 1 SD increase in log_e_-transformed HOMA2-IR within each SCORE2-Diabetes risk group. All models fulfilled the proportional hazards assumption, and model discrimination was assessed using Harrell’s C-index. We further investigated the net reclassification improvement when adding HOMA2-IR to SCORE2-Diabetes risk score using the low (<5%), intermediate (5–10%) and high risk (>10%) as cut-offs, testing the proportion of individuals who were correctly reclassified across risk categories relative to those who were incorrectly reclassified [29]. In addition, we evaluated the integrated discrimination improvement.

In an exploratory analysis, we also assessed whether addition of HOMA2-IR to the TIMI (Thrombolysis in Myocardial Infarction) Risk Score for Heart Failure in Diabetes (TRS-HF_DM_) improved prediction of hospitalisation for HF. TRS-HF_DM_ was originally developed for individuals with diabetes with and without prevalent HF [30].

All analyses were performed in STATA version 15.1 and R version 4.4.2.

## Results

### Baseline characteristics

A total of 19,892 ANDIS participants with newly diagnosed diabetes were included (median age at diagnosis 62.5 years, IQR 52.6–70.3; 58.6% male). Most participants were classified as having type 2 diabetes (91.1%; median age 63.0 years at diagnosis, 58.7% male), followed by LADA (4.6%; median age 60.9 years, 55.5% male), with smaller subsets of participants having type 1 diabetes (1.7%; median age 28.1 years, 58.1% male) or secondary diabetes (1.1%; median age 63.1 years, 62.9% male). Baseline characteristics for the overall study cohort and according to HF status are presented in Table 1, and characteristics according to diabetes subtype are presented in Table 2.

**Table 1:** Baseline descriptive characteristics for individuals with newly diagnosed diabetes by HF status

	No HF	Prevalent HF	Incident HF	Total
Sample size	18,149	819	924	*N*=19,892
Age at diagnosis (years)	61.6 (51.8–69.4)	71.2 (64.2–77.3)	69.8 (62.7–76.7)	62.5 (52.6–70.3)
Male sex	10,517 (57.9)	536 (65.4)	597 (64.6)	11,650 (58.6)
Length (cm)	171.9 ± 9.9	171.8 ± 9.9	172.1 ± 9.9	171.9 ± 9.9
Weight (kg)	91.3 ± 20.2	92.8 ± 21.5	92.2 ± 20.0	91.4 ± 20.3
BMI (kg/m^2^)	30.1 (26.7–34.1)	30.7 (27.1–34.9)	30.5 (27.1–34.3)	30.1 (26.8–34.2)
Obesity	9281 (51.1)	447 (54.6)	500 (54.1)	10,228 (51.4)
Fasting blood sugar (mmol/l)	7.2 (6.5–8.6)	7.4 (6.6–8.6)	7.5 (6.7–8.7)	7.3 (6.5–8.6)
HbA_1c_ (mmol/mol)	51.0 (45.0–71.0)	51.0 (46.0–61.0)	50.0 (45.0–66.0)	51.0 (45.0–70.0)
HbA_1c_ (%)	6.8 (6.3–8.6)	6.8 (6.4–7.7)	6.7 (6.3–8.2)	6.8 (6.3–8.6)
C-peptide (nmol/l)	1.1 (0.8–1.5)	1.5 (1.1–2.1)	1.2 (0.9–1.6)	1.1 (0.8–1.5)
GADA	1263 (7.0)	24 (2.9)	54 (5.8)	1341 (6.7)
HOMA2-%B	91.2 (63.3–121.3)	109.0 (82.6–145.8)	93.8 (65.1–125.4)	92.0 (64.0–122.0)
HOMA2-%S	34.4 (25.9–46.5)	25.6 (18.9–35.3)	31.4 (23.1–42.5)	34.0 (25.4–45.9)
HOMA2-IR	2.9 (2.2–3.9)	3.9 (2.8–5.3)	3.2 (2.4–4.3)	2.9 (2.2–3.9)
eGFR (ml/min per 1.73 m^2^)	95.2 (82.3–104.7)	80.6 (63.6–92.9)	85.4 (70.8–96.9)	94.3 (80.8–103.9)
eGFR <60 ml/min per 1.73 m^2^	767 (4.2)	159 (19.4)	115 (12.4)	1041 (5.2)
Treatments				
Dietary	7419 (40.9)	386 (47.1)	478 (51.7)	8283 (41.6)
Metformin	10,197 (56.2)	373 (45.5)	400 (43.3)	10,970 (55.1)
Insulin	1451 (8.0)	53 (6.5)	83 (9.0)	1587 (8.0)
Sulfonylurea	64 (0.4)	7 (0.9)	8 (0.9)	79 (0.4)
Diabetes type				
LADA	840 (4.6)	24 (2.9)	53 (5.7)	917 (4.6)
Secondary	208 (1.1)	7 (0.9)	9 (1.0)	224 (1.1)
Type 1	347 (1.9)	1 (0.1)	0 (0.0)	348 (1.7)
Type 2	16,486 (90.8)	785 (95.8)	856 (92.6)	18,127 (91.1)
Missing	268 (1.5)	2 (0.2)	6 (0.6)	276 (1.4)
Smoking (yes)	2008 (11.1)	61 (7.4)	111 (12.0)	2180 (11.0)
Systolic blood pressure (mmHg)	134 (125–142)	130.0 (120–140)	137.0 (127–145)	135 (125–144)
Diastolic blood pressure (mmHg)	80 (74–85)	75.0 (70–80)	80.0 (70–85)	80 (74–85)
Antihypertensive treatment	8099 (44.6)	520 (63.5)	537 (58.1)	9156 (46.0)
Lipid-lowering therapy	5756 (31.7)	367 (44.8)	372 (40.3)	6495 (32.7)

Data for categorical variables are *n* (%); those for continuous variables are means ± SD or median (IQR)

Obesity was defined as BMI ≥30 kg/m^2^

**Table 2:** Baseline descriptive characteristics for individuals with newly diagnosed diabetes by diabetes type

	LADA	Secondary diabetes	Type 1 diabetes	Type 2 diabetes
Sample size	917	224	348	18,127
Age at diagnosis (years)	60.9 (50.4–69.1)	63.1 (53.7–69.6)	28.1 (22.5–37.8)	63.0 (53.8–70.6)
Male sex	509 (55.5)	141 (62.9)	202 (58.0)	10,638 (58.7)
Length (cm)	172.4 ± 9.9	172.7 ± 9.1	175.4 ± 9.6	171.8 ± 9.9
Weight (kg)	86.1 ± 19.8	87.3 ± 21.3	70.5 ± 13.3	92.3 ± 20.0
BMI (kg/m^2^)	28.0 (24.8–32.1)	28.7 (25.1–33.2)	22.5 (20.2–25.1)	30.4 (27.1–34.4)
Fasting blood sugar (mmol/l)	7.9 (6.8–11.4)	7.4 (6.5–9.3)	11 (7.9–14)	7.2 (6.5–8.4)
HbA_1c_ (mmol/mol)	62 (48.9–94.0)	56.0 (47.0–79.1)	99.1 (82.4–115.8)	51.0 (45.0–68.0)
HbA_1c_ (%)	7.8 (6.6–10.8)	7.3 (6.5–9.4)	11.2 (9.7–12.7)	6.8 (6.3–8.4)
C-peptide (nmol/l)	0.8 (0.5–1.2)	1.0 (0.6–1.4)	0.2 (0.1–0.3)	1.2 (0.9–1.6)
GADA	904 (98.6)	11 (4.9)	348 (100)	0 (0)
HOMA2-%B	65.8 (32.0–98.7)	81.6 (43.6–119.1)	17.8 (10.1–34.7)	94.3 (67.9–124.1)
HOMA2-%S	44.0 (29.4–64.2)	36.0 (27.1–54.1)	121.4 (81.3–164.4)	33.2 (25.0–44.0)
HOMA2-IR	2.5 (1.7–3.4)	2.8 (1.9–3.7)	1.0 (0.7–2.3)	3.0 (2.3–4.0)
eGFR (ml/min per 1.73 m^2^)	95.2 (83.4–105.1)	95.5 (82.6–104.9)	119.7 (106.2–128.4)	93.4 (81.4–102.4)
Smoking (yes)	93 (10.1)	41 (18.3)	26 (7.5)	2000 (11.0)
Systolic blood pressure (mmHg)	131 (125–140)	132 (127–140)	127 (119–132)	132 (126–140)
Diastolic blood pressure (mmHg)	80 (75–82)	80 (72–82)	77 (70–80)	80 (75–84)
Antihypertensive treatment	366 (39.9)	112 (50.0)	31 (8.9)	9136 (50.4)
Lipid-lowering therapy	243 (26.5)	63 (28.1)	14 (4.0)	6195 (34.2)

Data for categorical variables are *n* (%); those for continuous variables are means ± SD or median (IQR)

### Incidence of HF and other outcomes

A total of 819 participants (4.1%) had prevalent HF at diabetes diagnosis, with a median time from HF to diabetes diagnosis of 3.5 years (IQR 6.7–1.3), as illustrated in ESM Fig. 1. These individuals were excluded from subsequent analyses of HF incidence and risk factors. Prevalent AMI before diabetes diagnosis was observed in 1235 participants (6.2%). Of the 819 participants with prevalent HF, 158 (19.3%) had previously been diagnosed with AMI. Individuals with prevalent AMI were excluded from analyses of incident non-ischaemic HF.

A total of 2198 (11.0%) participants died during up to 14 years of follow-up, with a median time to all-cause mortality of 5.1 years (IQR 2.3–8.1). Cardiovascular disease was the primary cause of death in 30% of individuals, and a contributing factor in 55.0% of participants (*n*=1209). Of the participants that died, 28.4% had prior HF, while 16.8% had had an AMI and 11.9% had a history of stroke.

During follow-up, a total of 924 participants (cumulative incidence 4.8%) were diagnosed with all-cause HF, during a median time from diabetes to all-cause HF of 4.1 years (IQR 1.7–6.9) and to non-ischaemic HF of 4.3 years (IQR 1.9–6.9; *n*=679). Incident AMI and stroke occurred in 554 (3.0%) and 567 (3.0%) of participants, respectively. Of all incident cardiovascular events, the first manifestation was most often atrial fibrillation (34%), followed by HF (28%), AMI (20%) and stroke (19%). The crude incidence rates (Table 3) of cardiovascular events increased with age. In the youngest age group, HF and AMI occurred at similar rates, with HF being slightly more frequent, whereas atrial fibrillation was more common in the older age groups.

**Table 3:** Incidence rates of cardiovascular outcomes by age group

	HF		AMI		Atrial fibrillation		Stroke	
Age group (years)	*n*	Incidence rate	*n*	Incidence rate	*n*	Incidence rate	*n*	Incidence rate
18–56	118	3.1 (2.6, 3.8)	110	3.0 (2.5, 3.6)	100	2.6 (2.2, 3.2)	87	2.3 (1.9, 2.8)
57–67	266	7.0 (6.2, 7.9)	222	6.0 (5.3, 6.9)	344	9.3 (8.4, 10.4)	169	4.4 (3.8, 5.1)
68–99	540	17.3 (15.9, 18.8)	222	6.9 (6.1, 7.9)	548	18.8 (17.3, 20.5)	306	9.3 (8.3, 10.4)

Values are incidence rates (95% CI) per 1000 person-years; *n* indicates the number of events within each age group (tertile)

Incidence rates increased with age for all outcomes (*p*<0.001)

The median age at the time of incident all-cause HF was 69.8 years (IQR 62.7–76.7). Male sex, higher BMI, lower eGFR, older age, use of lipid-lowering therapy and antihypertensive treatment were numerically more common among both prevalent and incident HF cases (Table 1).

### Risk factors

None of the relatively young participants with type 1 diabetes had incident HF during follow-up, but the cumulative HF incidence was similar in participants with type 2 diabetes (4.9%; median 4.1 years from diagnosis), LADA (5.9%; median 4.2 years from diagnosis) or secondary diabetes (4.1%; median 3.0 years from diagnosis). Risk factors were therefore only tested for participants with a diabetes type other than type 1.

Risk factors associated with all-cause and non-ischaemic HF are shown in Table 4. In age- and sex-adjusted models, the proportional hazards assumption was met according to the results of Schoenfeld residual tests for all covariates except for fasting blood sugar and HbA_1c_. However, use of Martingale residuals showed smooth, approximately linear, forms for both factors. The risk factors included in the age- and sex-adjusted models were sex, age, BMI, fasting blood sugar, HbA_1c_, C-peptide, GADA, insulin resistance (HOMA2-IR), systolic and diastolic blood pressure, use of lipid-lowering therapy, antihypertensive treatment, smoking and renal function. C-peptide was strongly correlated with HOMA2-IR. Conversely, HbA_1c_, GADA, systolic and diastolic blood pressure were not associated with HF.

**Table 4:** Risk factors for incident HF in the ANDIS cohort

	All-cause HF (*N*=924)		Non-ischaemic HF (*N*=676)	
	Age- and sex-adjusted	Full model	Age- and sex-adjusted	Full model
Age (1 year change)	1.08 (5.5 × 10^−119^)	1.08 (2.6 × 10^−81^)	1.07 (2.4 × 10^−80^)	1.08 (2.0 × 10^−57^)
Male sex	1.51 (2.7 × 10^–9^)	1.62 (6.5 × 10^−12^)	1.25 (4.5 × 10^–3^)	1.36 (1.2 × 10^–04^)
BMI (kg/m^2^)	1.05 (2.2 × 10^−17^)	1.04 (9.4 × 10^–9^)	1.06 (2.8 × 10^−22^)	1.06 (2.0 × 10^−14^)
Fasting blood sugar (mmol/l)	1.04 (1.0 × 10^–3^)	1.02 (8.0 × 10^–2^)	1.04 (1.8 × 10^–3^)	1.03 (2.6 × 10^–02^)
HbA_1c_ (10 × mmol/mol)	1.00 (3.6 × 10^–1^)	–	1.00 (3.3 × 10^–1^)	–
C-peptide (nmol/l)	1.51 (1.9 × 10^−22^)	–	1.45 (2.5 × 10^−13^)	–
GADA	1.00 (3.3 × 10^–1^)	–	1.00 (5.4 × 10^–1^)	–
HOMA2-IR (log_e_, per 1 SD increase)	1.35 (1.0 × 10^−19^)	1.18 (9.0 × 10^–5^)	1.33 (2.6 × 10^−14^)	1.11 (3.2 × 10^–02^)
eGFR (10 × ml/min per 1.73 m^2^)	0.92 (6.3 × 10^–5^)	0.94 (2.6 × 10^–3^)	0.92 (3.1 × 10^–4^)	0.93 (4.9 × 10^–03^)
Systolic blood pressure (mmHg)	1.00 (8.3 × 10^–1^)	–	1.00 (3.5 × 10^–1^)	–
Diastolic blood pressure (mmHg)	1.00 (3.6 × 10^–1^)	–	1.01 (1.1 × 10^–1^)	–
Antihypertensive treatment	1.26 (8.9 × 10^–4^)	1.09 (2.3 × 10^–1^)	1.23 (8.5 × 10^–3^)	1.12 (1.7 × 10^–01^)
Lipid-lowering therapy	1.25 (1.0 × 10^–3^)	1.14 (7.0 × 10^–2^)	1.10 (2.5 × 10^–1^)	–
Smoking	1.60 (4.7 × 10^–6^)	1.60 (4.6 × 10^–6^)	1.45 (2.3 × 10^–3^)	1.50 (9.6 × 10^–04^)
Diabetes type				
Type 2	Reference	–	Reference	–
LADA	1.05 (7.4 × 10^–1^)		0.99 (9.4 × 10^–1^)	
Secondary	0.93 (8.3 × 10^–1^)		0.99 (9.7 × 10^–1^)	

Values are HRs and *p* values from Cox proportional hazards regression models in individuals with newly diagnosed diabetes other than type 1 who were free from diagnosed HF at baseline. Survival times were defined as time from baseline to HF diagnosis. Covariates in the full, multivariable-adjusted models were based on significant predictors from age and sex-adjusted models in which HOMA2-IR was selected instead of the highly correlated plasma C-peptide concentration

Variables not significant in sex- and age-adjusted models are indicated with a dash in the full model column since they were not included. C-peptide is marked with a dash due to collinearity with HOMA2-IR (as mentioned in the text)

The risk factors significantly associated with incident HF in the age- and sex-adjusted models were then tested in multivariable-adjusted models. Risk factors that remained independently associated with HF in these models were male sex, higher age at onset, BMI, HOMA2-IR, smoking and renal function. The association between HOMA2-IR and HF was consistent across age tertiles, with no evidence of effect modification by age (*p*_interaction_=0.57). Fasting glucose, use of lipid-lowering therapy and antihypertensive treatment were not independently associated. All multivariable models met the proportional hazards assumption, with concordant results found in analyses with and without imputation (data not shown).

**Fig. 1:**
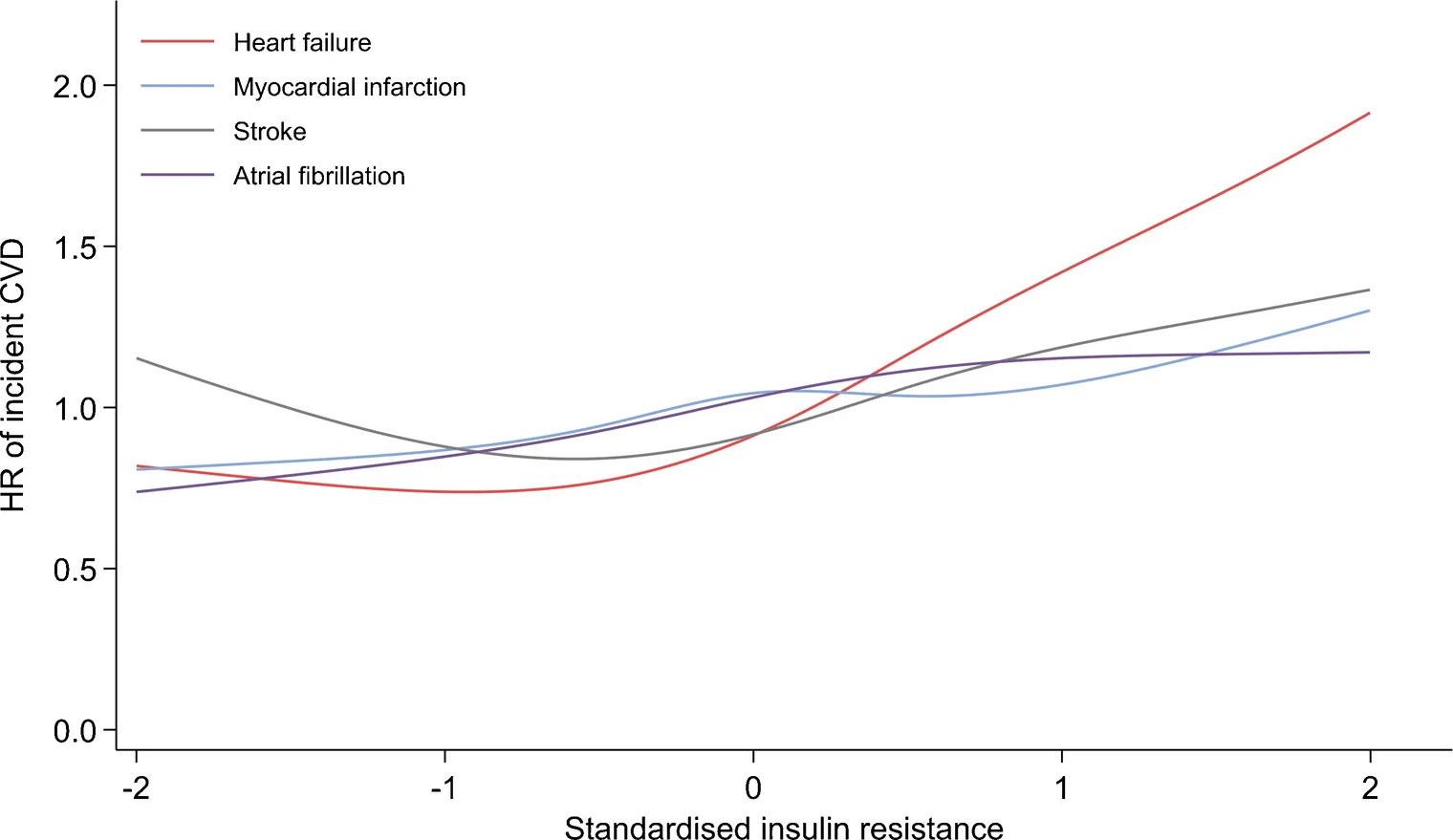
Risk of incident cardiovascular disease in relation to insulin resistance. Restricted cubic spline analysis indicating the HRs for incident cardiovascular diseases (CVD) using Cox proportional hazards regression models adjusted for age and sex. Standardised insulin resistance is centred around the mean, and modelled across five knots as recommended by Harrell et al [28]

Given that HOMA2-IR, rather than glucose measures, was strongly and independently associated with incident HF, we examined cumulative HF incidence in relation to HOMA2-IR, fasting glucose and HbA_1c_ using non-linear models. First, in quartile analyses, the risk of both incident all-cause and non-ischaemic HF increased with higher HOMA2-IR (Q3–Q4 vs Q1, *p*<0.05; ESM Fig. 2). For fasting glucose, only Q4 was associated with all-cause HF (HR 1.3; 95% CI 1.07, 1.56), with no association for non-ischaemic HF. Similarly, no association was seen with HbA_1c_. Second, in age- and sex-adjusted cubic spline analyses, HOMA2-IR showed the strongest association with incident HF compared with incident AMI, stroke and atrial fibrillation (Fig. 1), independent of BMI. For fasting blood sugar, we observed marked variation in HRs for HF at low blood sugar levels when using five knots, which suggested overfitting, and therefore used three knots instead (placed at the 10th, 50th and 90th percentiles). We detected no non-linear associations between glucose-related covariates and risk of incident HF (ESM Fig. 3). Finally, given the strong association of HOMA2-IR with obesity, we examined the association of obesity with HF, and found associations both with all-cause HF (HR 1.54; 95% CI 1.35, 1.76; *p*=1.3 × 10^−10^) and non-ischaemic HF (HR 1.85; 95% CI 1.58, 2.16; *p*=9.8 × 10^−15^) in age- and sex-adjusted models, compared with non-obese individuals (ESM Fig. 4).

### HF risk prediction using the SCORE2-Diabetes risk score and insulin resistance

After excluding individuals <40 years old and those with prevalent AMI or stroke, the cohort comprised 15,891 individuals. In this SCORE2-Diabetes population, the moderate-risk group had an increased risk of incident HF (HR 2.27; 95% CI 1.24, 4.16; *p*=7.8 × 10^−3^) compared with the low-risk group (reference), as did the high-risk group (HR 5.70; 95% CI 3.21, 10.12; *p*=3.0 × 10^−9^) and the very-high-risk group (HR 15.00; 95% CI 8.41, 26.77; *p*=4.7 × 10^−20^; ESM Fig. 5). Overall discrimination of incident all-cause HF, as assessed by Harrell’s C-index, was 0.68.

**Fig. 2:**
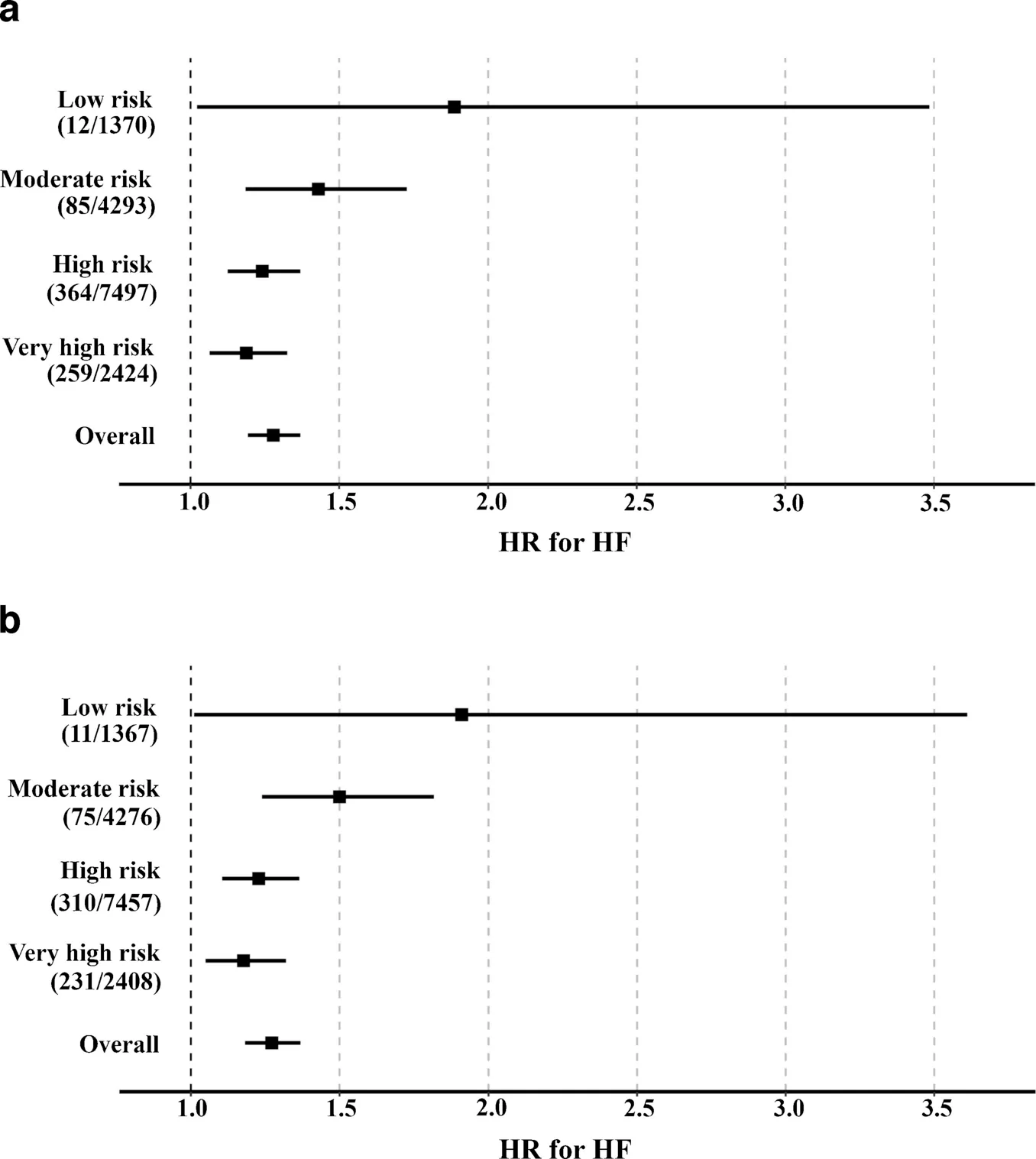
Risk of HF in relation to increasing HOMA2-IR across SCORE2-Diabetes risk groups. Plots show the HR per 1 SD increase in log_e_-transformed HOMA2-IR within each SCORE2-Diabetes risk category. Risk of (**a**) incident all-cause HF and (**b**) non-ischaemic HF. The numbers in parentheses indicate incident HF cases and the total number of participants included in Cox proportional hazard regression analyses. Totals may differ from the overall SCORE2-Diabetes cohort (*n*=15,891) as 307 participants (all-cause HF) and 383 participants (non-ischaemic HF) were excluded due to lack of follow-up data. Risk is expressed as HRs with 95% CI

Insulin resistance (as assessed using HOMA2-IR) predicted incident all-cause HF across all SCORE2-Diabetes risk groups (Fig. 2). The HR per 1 SD increase in HOMA2-IR was highest in the low-risk group (HR 1.89; 95% CI 1.02, 3.48; *p*=4.3 × 10^−2^) and lowest in the very-high-risk group (HR 1.19; 95% CI 1.06, 1.32; *p*=2.2 × 10^−3^). Overall, adding HOMA2-IR to the SCORE2-Diabetes risk score resulted in a modest increase in the C-index (0.71), with concordant results for non-ischaemic HF (ESM Table 1). The integrated discrimination improvement metric improved significantly (*p*<0.001). Adding HOMA2-IR yielded a net reclassification improvement of 0.06 (6%, *p*<0.001), driven by a 12.5% upward reclassification among individuals who developed HF. In exploratory analyses, log_e_-transformed, standardised urinary albumin–creatinine ratio (imputed using random forest methods due to >50% missingness) was independently associated with incident all-cause HF after adjustment for the SCORE2-Diabetes risk score and HOMA2-IR (HR 2.01 per SD; 95% CI 1.90, 2.13; *p*<0.001). HOMA2-IR also remained independently associated with HF hospitalisation after adjustment for TRS-HF_DM_ (HR 1.18 per SD; 95% CI 1.10, 1.26; *p*<0.001), with the C-index again increasing from 0.68 to 0.71. The integrated discrimination improvement metric also improved (*p*<0.05), whereas net reclassification improvement was not significant (−0.004, *p*=0.52), mainly due to minimal reclassification and few individuals being in the highest risk category.

## Discussion

The three main findings of the current study were the high incidence of non-ischaemic HF over a median follow-up of 5 years in individuals with newly diagnosed diabetes, the finding of insulin resistance assessed by HOMA2-IR and low renal function being the main risk factors for such early non-ischaemic HF beyond conventional risk factors, and the finding that insulin resistance adds incremental predictive value to the SCORE2-Diabetes risk score.

The cumulative incidence of HF was 60% higher than for incident AMI and stroke, and the overlap with vascular disease was limited, with a preceding AMI observed in only one quarter of incident HF cases. HF prevalence was also more common than vascular outcomes in individuals with diabetes who died. Our findings thus highlight non-ischaemic HF as a common diabetes complication with relatively early onset after diabetes diagnosis that is also associated with mortality. These observations are consistent with a previous report from administrative UK registers, in which HF and peripheral arterial disease were the most common initial cardiovascular manifestations of diabetes [31], as well as multinational data demonstrating that HF and chronic kidney disease are early complications of type 2 diabetes that are associated with increased mortality [32]. We also observed a high cumulative incidence of atrial fibrillation, which was not assessed in the UK study. The cumulative incidence of HF in the UK study (14.1%) was almost three times higher than in our cohort, despite a similar follow-up time (median 5.5 years) and baseline age. However, our study included all new clinically diagnosed consecutive cases of diabetes whereas the UK study applied several exclusions that may have influenced estimates, including those aged <30 years, those registered for <1 year in a healthcare practice that met research data recording standards, and those with diabetes of uncertain type. A study of individuals with type 2 diabetes from the American Kaiser Permanente healthcare system in the 1990s, with a median age similar to that in our study, found an HF incidence of 7.7% over just 30 months of follow-up [13]. However, the median time from diagnosis exceeded 5 years, 40% had ischaemic heart disease, and both the diagnostic definitions and therapeutic strategies for diabetes and cardiovascular disease have changed markedly since then. Nevertheless, the high prevalence and incidence of HF in relation to diabetes diagnosis – and the disconnect with cardiovascular risk factors as reported previously [2] – suggest contributions from risk factors that are not routinely assessed in clinical practice.

One such risk factor, insulin resistance as assessed by HOMA2-IR, was independently associated with incident HF in our study. Although associations between insulin resistance and incident HF have previously been reported in studies of individuals with diabetes [33, 34] and those without [35, 36], large studies comprising all individuals consecutively diagnosed with diabetes at specific timepoints such as diagnosis have been lacking. In 2005, two studies by Ingelsson et al [34, 37] demonstrated that insulin resistance, measured by proinsulin and clamp glucose disposal rate, independently predicted incident HF in middle-aged and older men. Since then, insulin resistance has been shown to be associated with all-cause HF and death [33] in individuals with newly diagnosed diabetes, although data on whether HF was ischaemic or non-ischaemic were not available in that study. An association between higher fasting insulin levels and both ischaemic and non-ischaemic HF has been observed in a study of older individuals without diabetes at baseline [35]. In the community-based ARIC cohort [36], insulin resistance displayed a non-linear relationship with incident all-cause HF in individuals without diabetes, but HF subtype was not recorded. In a recent study of individuals with newly diagnosed type 2 diabetes, increased C-peptide values were found to be associated with increased risk of cardiovascular events, but HF was not included [38]. In the present study of all individuals consecutively diagnosed with diabetes, we also found an association with incident all-cause and non-ischaemic HF for both insulin resistance and C-peptide levels. Furthermore, insulin resistance provided independent predictive value beyond the SCORE2-Diabetes risk score, and added incremental discrimination to the risk model, as well as net reclassification improvement. The association between insulin resistance and all-cause and non-ischaemic HF was strongest in low-risk individuals, and gradually attenuated across higher SCORE2-Diabetes risk groups, but remained a robust, independent and statistically significant predictor of HF. The distribution across SCORE2-Diabetes categories and corresponding HRs for HF were comparable to those reported by Karayiannides et al [19]. Whereas the SCORE2-Diabetes risk score only predicted all-cause HF in that study, we observed similar associations for both all-cause and non-ischaemic HF.

In exploratory analyses, insulin resistance remained independently associated with HF hospitalisation after adjustment for the TRS-HF_DM_ score. However, these findings should be interpreted with caution, as TRS-HF_DM_ was developed in populations of individuals with type 2 diabetes and established cardiovascular disease or multiple cardiovascular risk factors [39], whereas our cohort comprised individuals with newly diagnosed diabetes with a lower burden of cardiovascular comorbidity.

Surprisingly, measures of glucose disturbance were not strongly associated with HF incidence. HbA_1c_ showed no association with HF incidence, and fasting glucose was only weakly associated, with the association being driven by the highest glucose quartile. In a previous study of HbA_1c_ [40], suboptimal glucose management (HbA_1c_ >7.0% [53 mmol/mol]) was associated with a higher risk of HF hospitalisation. Similarly, in the Go-DARTS cohort, high levels of HbA_1c_ (>10%), as well as low levels (<6%), predicted the development of incident HF [41]. As we did not have access to serial samples, we were unable to reproduce these results. Nevertheless, our study suggests that, at diabetes diagnosis, measures of insulin resistance are more strongly predictive of HF than measures of glucose disturbance. Previous population-based studies with single HbA_1c_ measures found an association of HbA_1c_ with incident HF [42, 43], but not when adjusting for diabetes [42] or when restricting analysis to recently diagnosed diabetes cases [43], consistent with our findings. Impaired fasting glucose levels (5.6−6.9 mmol/l) in individuals without diabetes have been associated with future HF [44, 45], and one study demonstrated a strong linear association between fasting glucose levels and incident HF in high-risk individuals with diabetes [46]. However, that study largely included individuals with established cardiovascular disease, which was not present in our study. It is reasonable to assume that a single baseline value for glucose and HbA_1c_ does not necessarily reflect future dysregulated diabetes and risk of incident HF in a linear fashion, whereas C-peptide levels may reflect more profound metabolic perturbation. Individuals with the highest blood sugar values at baseline may also represent a more treatment-resistant subgroup who are at increased HF risk.

Several limitations need to be addressed. First, we were unable to fully account for the effects of glucose-lowering and/or other treatments. At the time of the analyses, only information about medications at baseline was available, which limits conclusions regarding incident HF and treatment initiation, discontinuation of treatments or dose modifications during follow-up. Given the dates between which the study data were collected (2008–2021), few participants were likely to have received treatment with the more recently introduced glucagon-like peptide-1 (GLP-1) receptor analogues and sodium–glucose cotransporter-2 (SGLT2) inhibitors. As these drugs confer beneficial effects on the risk factors identified here (insulin resistance and renal function), future studies on the impact of these drugs on HF burden will be of substantial interest. Second, only a single blood sample at diabetes diagnosis was available. Given the pulsatile nature of insulin secretion, the availability of repeated C-peptide measurements would probably improve the reliability and interpretation of HOMA2-derived insulin resistance estimates. However, for cohort and epidemiological studies, the challenges of repeated sampling make HOMA2 a widely accepted tool for assessment of insulin resistance [47]. HOMA2 further accounts for the non-linear relationship between fasting glucose and insulin/C-peptide, providing a more physiologically accurate estimate of insulin resistance than HOMA1. Other biomarkers of insulin resistance, such as the triglyceride–glucose (TyG) index [48], were not available in our dataset. Third, risk factors for HF in type 1 diabetes could not be examined, as no incident HF occurred during follow-up, probably due to the young age of these individuals and limited follow-up duration. Fourth, we cannot rule out the possibility that some individuals with non-ischaemic HF actually had ischaemic HF. Silent myocardial ischaemia is more common in individuals with diabetes due to autonomic neuropathy, and has been reported to occur in 1 in 5 individuals with diabetes [49]. Fifth, sex was included as an adjustment variable in all analyses. However, sex-stratified analyses were not performed, and potential differences in risk factor associations by sex were therefore not assessed.

In conclusion, we report a high incidence of HF over a median of 5 years after diabetes diagnosis, for which insulin resistance and low renal function were the main risk factors, in addition to conventional targets for disease prevention. Our findings suggest an important role for cardiac assessment in individuals with newly diagnosed diabetes, particularly in the presence of insulin resistance as measured by elevated HOMA2-IR. Importantly, our findings also demonstrate that insulin resistance provides incremental predictive value when added to the SCORE2-Diabetes risk score, underscoring its relevance in early HF risk stratification and supporting the value of assessing HOMA2-IR in clinical practice. The impact of the more recently introduced SGLT2 inhibitors and GLP-1 receptor agonists, which confer beneficial effects on these risk factors, on HF incidence in the general diabetes population soon after diagnosis will require further study.

## Supplementary Information

Below is the link to the electronic supplementary material.

ESM (PDF 479 KB)

## Data Availability

Individual-level data are not publicly available due to ethical and legal restrictions. De-identified data are available upon reasonable request after publication, with approval from the ANDIS steering committee.

## Abbreviations

AMI: Acute myocardial infarction
ANDIS: All New Diabetics in Scania
HF: Heart failure
LADA: Latent autoimmune diabetes in adults
NDR: Swedish National Diabetes Register
SCORE2-Diabetes: Systematic Coronary Risk Evaluation 2-Diabetes
