# Supplementary material for: Incidence and independent risk factors for heart failure in a population-based cohort of 19,892 individuals with newly diagnosed diabetes
Source: Diabetologia. 2026 Aug 3;69(11):3248–60. doi: 10.1007/s00125-026-06823-1 (PMC13627193; doi:10.1007/s00125-026-06823-1)
Supplement: Supplementary file 1 [file 125_2026_6823_MOESM1_ESM.pdf]

Electronic supplementary material (ESM)

ESM Table 1. SCORE2-Diabetes and insulin resistance as predictors of incident HF.

SCORE2-Diabetes risk group	All-cause HF				Non-ischaemic HF			
	SCORE2-Diabetes		+ HOMA2-IR		SCORE2-Diabetes		+ HOMA2-IR	
	HR (95% CI)	*p*-value	HR (95% CI)	*p*-value	HR (95% CI)	*p*-value	HR (95% CI)	*p*-value
Low (reference)	1.0	-	1.89 (1.02, 3.48)	$4.3x10^{-02}$	1.0	-	1.91 (1.01, 3.61)	$4.6x10^{-02}$
Moderate	2.27 (1.24, 4.16)	$7.8x10^{-03}$	1.43 (1.18, 1.73)	$2.0x10^{-04}$	2.19 (1.16, 4.12)	$1.5x10^{-02}$	1.50 (1.24, 1.82)	$3.4x10^{-05}$
High	5.70 (3.21, 10.12)	$3.0x10^{-09}$	1.24 (1.12, 1.37)	$1.7x10^{-05}$	5.18 (2.84, 9.45)	$8.3x10^{-08}$	1.23 (1.10, 1.36)	$1.4x10^{-04}$
Very high	15.00 (8.41, 26.77)	$4.7x10^{-20}$	1.19 (1.06, 1.32)	$2.2x10^{-03}$	12.96 (7.08, 23.73)	$1.0x10^{-16}$	1.18 (1.05, 1.32)	$5.5x10^{-03}$
Harrell's C-index	0.68		0.71		0.68		0.71	

HR with 95% CIs for all-cause and non-ischaemic HF are shown for SCORE2-Diabetes risk groups (univariate models) and per 1 standard deviation (SD) increase in log-transformed HOMA2-IR within each SCORE2-Diabetes risk group (multivariable models). Harrell's C-indices are reported for each model. SCORE2-Diabetes, Systematic Coronary Risk Evaluation 2-Diabetes

ESM Fig. 1 Temporal relation of cumulative HF incidence to diabetes diagnosis.

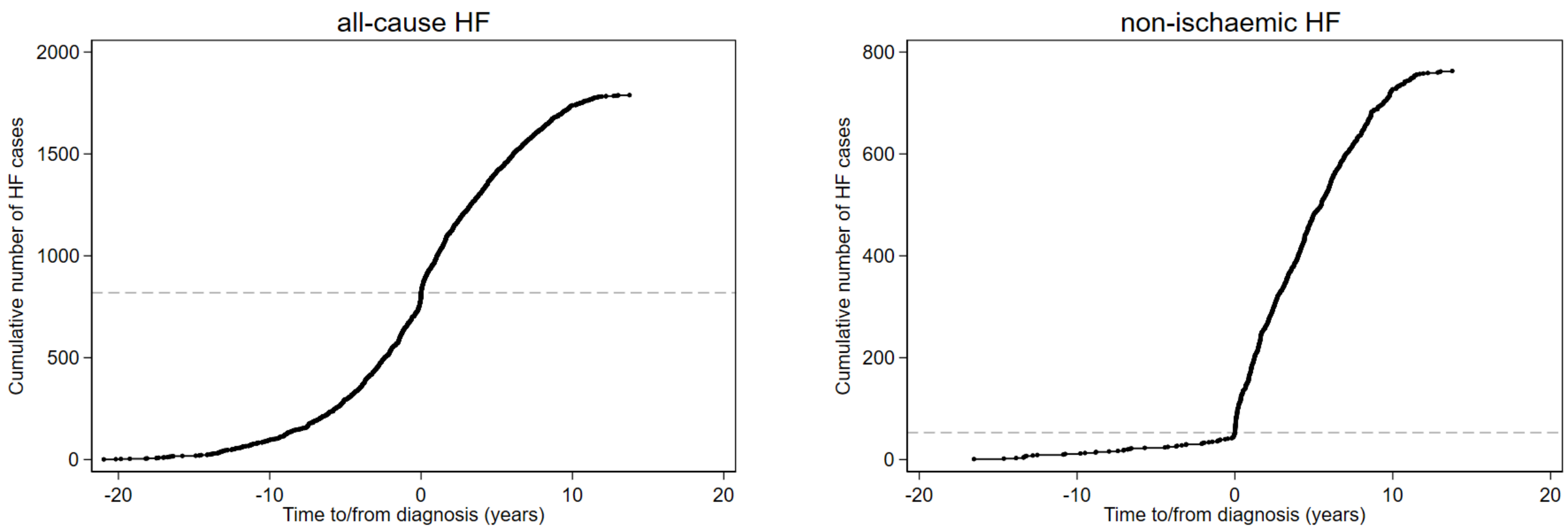

ESM Fig. 2 Quartiles of insulin resistance and risk of incident HF.

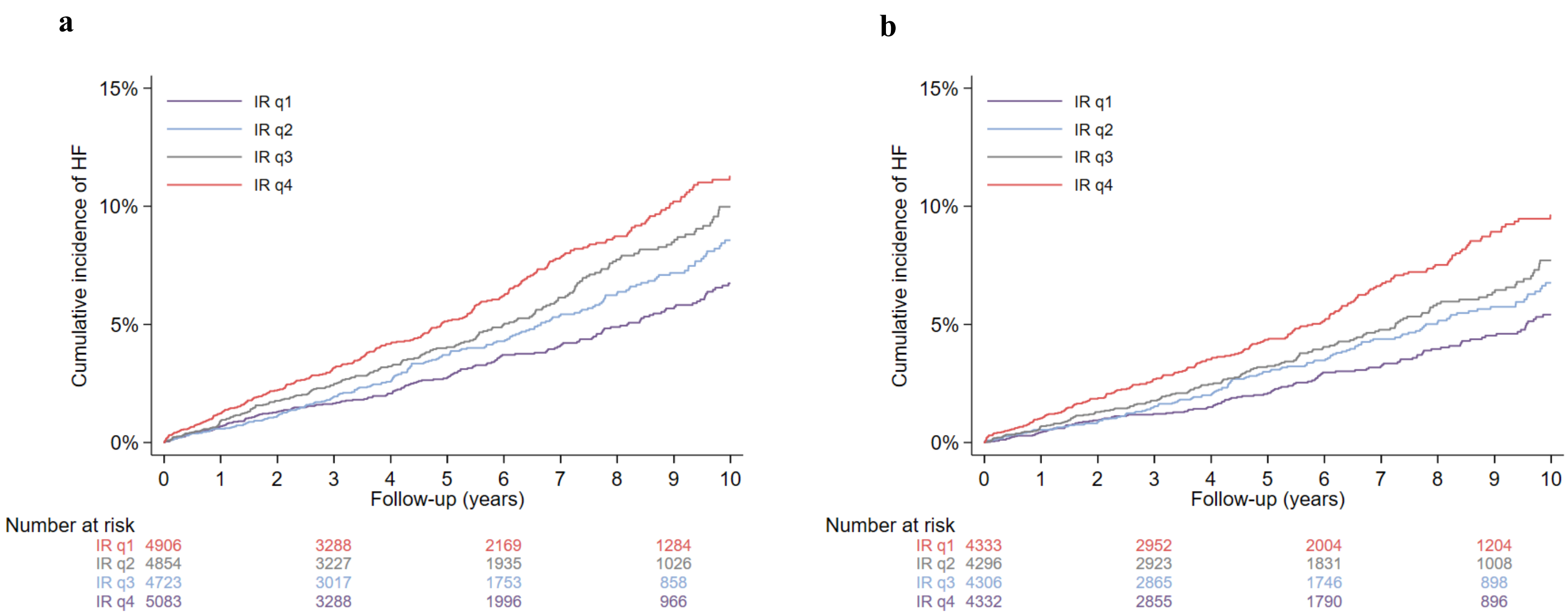

Cumulative incidence of incident heart failure (HF) by quartiles of insulin resistance (IR), during 10 years of follow-up. All-cause HF (**a**) and non-ischaemic HF (**b**)

ESM Fig. 3 Cubic spline analyses of glucose-related risk factors and incident HF.

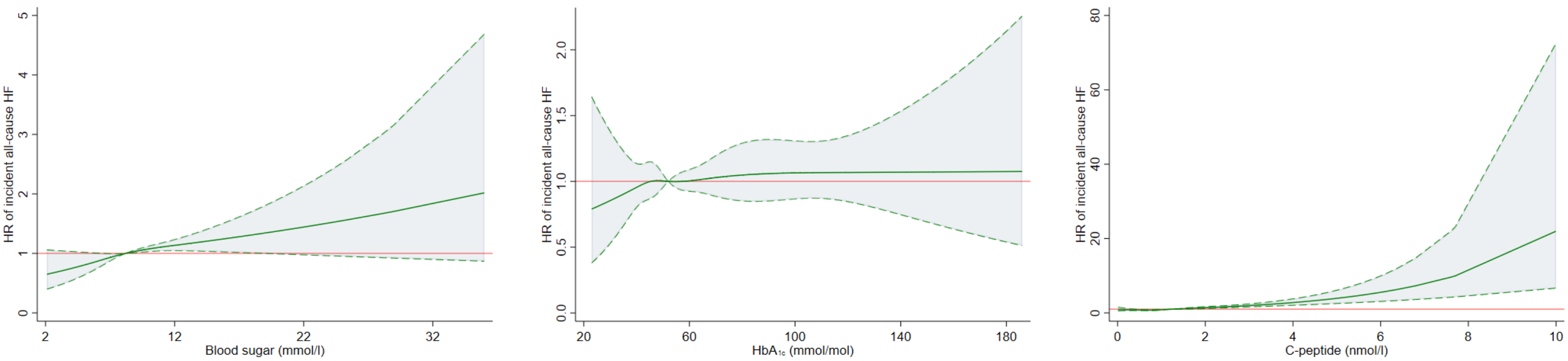

Restricted cubic splines of glucose-related risk factors (fasting blood sugar, HbA_{1c}, C-peptide) adjusted for age and gender, and HR of incident all-cause HF in Cox proportional hazard regression models. Risk factors are centered around the mean and with knots placed as proposed by Harrell. Test for non-linear associations: bloodsugar (3 knots), p=0,36; Hba1c (5 knots), p=0,95; C-peptide (5 knots), p=0,26.

ESM Fig. 4 Obesity and risk of incident all-cause and non-ischaemic HF.

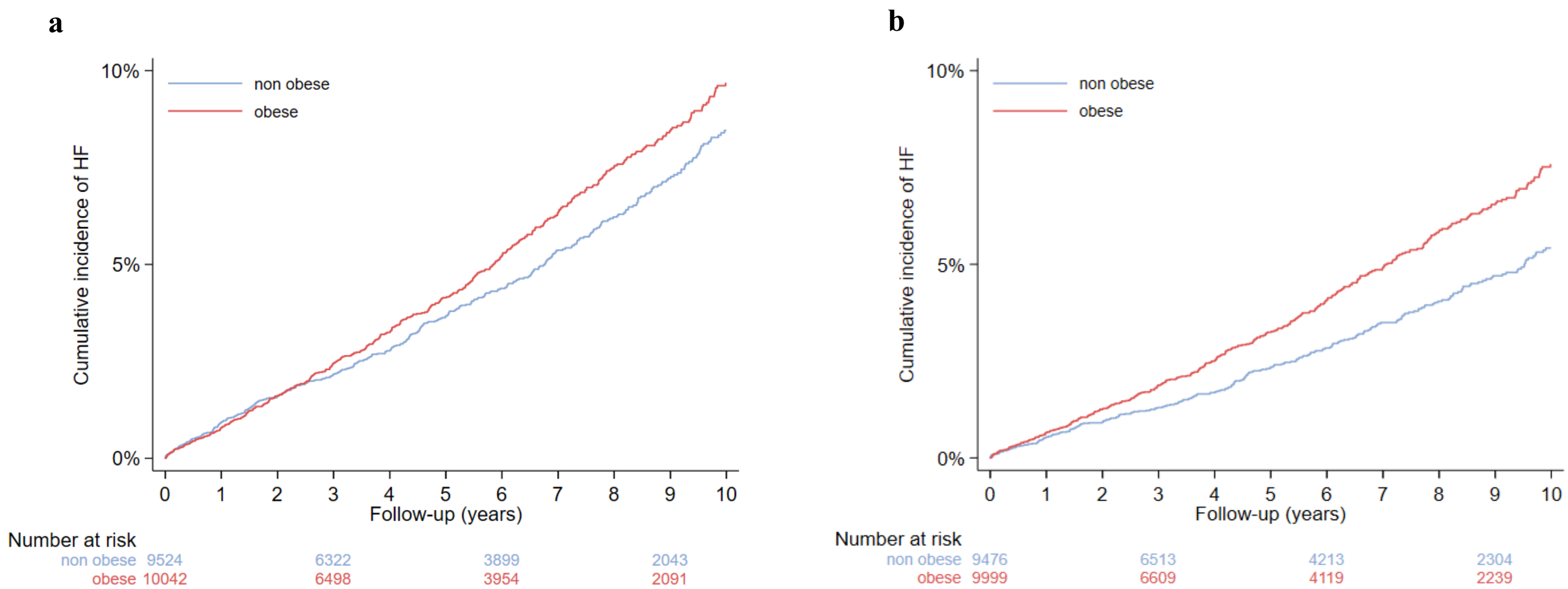

Cumulative incidence of incident HF by obesity status, during a period of 10 year follow-up. All-cause HF (**a**) and non-ischaemic HF (**b**).

ESM Fig. 5 Hazard ratios for HF across SCORE2-Diabetes groups

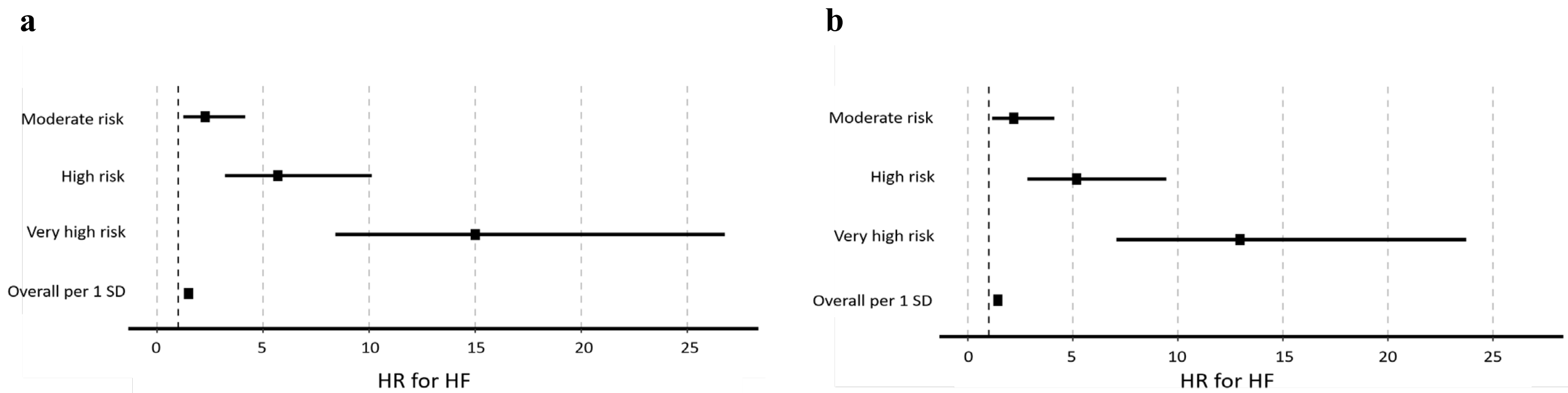

HRs with 95% CIs for all-cause HF (**a**) and non-ischaemic HF (**b**) across SCORE2-Diabetes moderate, high, and very-high-risk groups, with the low-risk group as the reference. The overall continuous SCORE2-Diabetes score effect is expressed per 1 SD (*z*-standardised) increase, derived from Cox proportional hazards models.
